# Particle Number Concentration Measurements on Public Transport in Bangkok, Thailand

**DOI:** 10.3390/ijerph20075316

**Published:** 2023-03-29

**Authors:** James C. Matthews, Chalida Chompoobut, Panida Navasumrit, M. Anwar H. Khan, Matthew D. Wright, Mathuros Ruchirawat, Dudley E. Shallcross

**Affiliations:** 1 Atmospheric Chemistry Research Group, School of Chemistry, University of Bristol, Cantock’s Close, Bristol BS8 1TS, UK; 2 Chulabhorn Research Institute, 54 Kamphaeng-Phet 6 Road, Laksi, Bangkok 10210, Thailand; 3 Department of Chemistry, University of the Western Cape, Robert Sobukwe Road, Bellville 7375, South Africa

**Keywords:** particulate matter, aerosol, ultrafine particles, personal exposure, public transport, bus, train, Skytrain, underground

## Abstract

Traffic is a major source of particulate pollution in large cities, and particulate matter (PM) level in Bangkok often exceeds the World Health Organisation limits. While PM_2.5_ and PM_10_ are both measured in Bangkok regularly, the sub-micron range of PM, of specific interest in regard to possible adverse health effects, is very limited. In the study, particle number concentration (PNC) was measured on public transport in Bangkok. A travel route through Bangkok using the state railway, the mass rapid transport underground system, the Bangkok Mass Transit System (BTS) Skytrain and public buses on the road network, with walking routes between, was taken whilst measuring particle levels with a hand-held concentration particle counter. The route was repeated 19 times covering different seasons during either morning or evening rush hours. The highest particle concentrations were found on the state railway, followed by the bus, the BTS Skytrain and the MRT underground with measured peaks of 350,000, 330,000, 33,000 and 9000 cm^−3^, respectively, though particle numbers over 100,000 cm^−3^ may be an underestimation due to undercounting in the instrument. Inside each form of public transport, particle numbers would peak when stopping to collect passengers (doors opening) and decay with a half-life between 2 and 3 min. There was a weak correlation between particle concentration on bus, train and BTS and Skytrain with carbon monoxide concentration, as measured at a fixed location in the city.

## 1. Introduction

Particulate matter (PM) is a known health risk, with some evidence of particulates < 2.5 µm in size being associated with cancers [1,2] and other diseases, e.g., heart disease [3,4] and diabetes [5,6]. The International Agency for Research on Cancer (IARC) [7] classified both pollutants as a whole, and PM specifically, as class 1 carcinogens. Pollutants in this context include any substance in the air that modify the atmosphere [8]. A number of studies (e.g., [9,10,11,12]) found a statistically significant association between PM_10_ and lung cancers, while some other studies (e.g., [13,14,15,16,17]) found a statistically significant link with PM_2.5_ and lung cancers. In Bangkok, local measurements of PM_2.5_ and PM_10_ regularly exceed the World Health Organisation (WHO) and Thai National Ambient Air Quality Standards (NAAQS) daily and annual limits due to high traffic levels [18,19], which may have a significant impact on human health.

While the adverse health impact of PM is proven and the larger size fractions of PM in terms of mass are well characterised, there is a need to provide more measurements of particle number concentrations (PNC) including the sub-micron and ultrafine ranges [20,21]. Dionisio et al. [22] found that central site monitoring may produce better exposure estimates for pollutants that are homogenously distributed such as PM_10_, but are less appropriate for more heterogeneous pollutants such as the ultrafine size fraction. Kumar et al. [23] reviewed measurements of nanoparticles in cities and recommended personal monitoring due to the difficulty in assessing exposure from fixed measurements. Lavigne et al. [24] linked residential ultrafine particle exposure to childhood cancer with a significant increase in risk with increasing exposure to PNC above 10,000 cm^−3^, observing an increased hazard risk of childhood cancer with increasing exposure of the mother to PNC within the first trimester of pregnancy. 

A recent PNC measurement from a fixed location on the 5th floor of a building in Lak Si, Bangkok, Thailand, in March 2018 indicated a diurnal cycle related to traffic emissions, with a maximum between 6:00 and 8:00 local time, and a second peak after 18:00 [25,26]. Peak concentrations were higher in roadside spot measurements [26]. In another study in Copenhagen, kerbside measurements showed elevated PNC, and the near-road exposure to pollutants was significantly higher than other exposed areas [27]. Karner et al. [28] demonstrated that some particulate matter, when measured as PNC, showed a 50% decrease in concentration at 15 m from the road, while particulate matter measured by mass (e.g., larger size fractions) showed no trend with distance. Road measurements of PNC are variable temporally and spatially as they are subject to road conditions and traffic behaviour, such as acceleration [29]. In measurements in Xi’an, China, the variation in roadside ultrafine particle concentration was affected by traffic volume and the meteorology [30].

Pongpiachan and Iijima [31] measured metals in Bangkok within PM_10_, but recent particle-size-segregated measurements in Bangkok detected metallic content within particles < 200 nm in size [26,32]. Maher et al. [33] found metallic particles < 200 nm within the brains of deceased children; these could have entered the brain through air pollution, which supports the hypothesis of the carcinogenicity of submicron or ultrafine particles.

Traveller exposures to pollutants differ by transport mode and fuel type in several previous studies. It is well established from a range of studies that vehicle passengers have a higher exposure to pollutants, including ultrafine particles, than pedestrians [34,35,36,37,38,39]. However, when travel time is considered, the inhaled dose may be higher in pedestrians and cyclists [40,41]. 

Jinsart et al. [42] took measurements of PM on four bus routes in Bangkok, including the route in this study, and showed that the concentrations of PM_10_ and PM_2.5_ in January (cool season) were significantly higher than that in August (rainy season). The concentrations were highest for both PM_10_ and PM_2.5_ in non-air-conditioned buses compared with air-conditioned buses. PM_2.5_ concentrations were compared in bus journeys taken in Singapore and Danang (Vietnam), two cities with similar climates but different socio-economic conditions, which resulted in higher concentrations in Vietnam [43]. Chaudhry and Elumalai [44] measured PNC within school buses in Dhanbad city, India, and showed elevated concentrations when windows were open as opposed to being closed. Exchange rates increased with journey speed, which is consistent with previous tracer studies for vehicles [45]. The ventilation condition and activity within a bus can affect particle size distribution [37,44]. Pollutants were found to be higher in diesel buses than in electric buses [39].

Electric trains can produce metal-rich ultrafine particles from brake linings, friction between the wheels and rail and from overhead pantographs [46]. However, diesel trains are very large contributors to ultrafine particles, more so than electric trains [47]. Emissions from diesel trains include both combustion sources and abrasive sources, which can differ in chemical composition and morphology [48]. Exposures inside the carriages are more often than that measured outside when the train operates in pull mode as opposed to push mode [48,49,50]. Modern underground train systems, such as the mass rapid transport (MRT) in Bangkok, do not have combustion engines, so all particle emissions are caused mainly by abrasion or resuspension; however, particle compositions, and hence, toxicology, can vary between different metro systems [51].

In Bangkok, traffic is often congested and efforts are being made to improve transportation around the city [52]. The Mass Transit Master Plan in the Bangkok Metropolitan Region (M-MAP) acts as a master plan for all aspects of the rail transit system including environmental impacts and public engagement. The plan is controlled by the Office of Transport and Traffic Policy and Planning (OTP) of the Ministry of Transport. Transit types of the Bangkok Metropolitan Region are: 1. Rapid transit consists of heavy rail (metro and commuter rail) including both the MRT and BTS systems lines; 2. Light rail (monorail and people mover) lines with 8 working lines (total planned: 15 lines). The system length has a planned total of 540 km (340 mi) in 2029, whereas service length is 210.25 km (130.64 mi), with 123 km (76 mi) under construction in 2021 [53].

The number of passengers taking public transport in Bangkok in 2018 has increased from previous years [53]. In 2018, 37,993,567 people used the train (including 1st, 2nd, 3rd class and Mae Klong); a total of 131,355,923 people used the MRT underground (both blue and purple lines) and 240,139,471 people used the BTS Skytrain. The traffic information network reported that in 2019, there were 25 million trips per year by bus [54].

The reasons for journeys in Bangkok are provided in a study by Witchayaphong et al. [55]. These were predominantly work (81%), school (8%), business (6%) and shopping (5%). Travel time on mass transit was less than 30 min for 31% of commuters, between 30 and 60 min for the majority (58%) and more than 60 min for 4%. It may be assumed that state railway journeys are longer than the MRT, BTS and bus journeys. Road-based transport usage is expected to increase, and the emissions of common pollutants (e.g., carbon dioxide (CO_2_), carbon monoxide (CO), nitric oxide (NO), nitrogen dioxide (NO_2_), and PM) were projected by Cheewaphongphan et al. [56] to fall from 2015 until 2022 and then rise again slightly until 2050; this is due to the competing effects of current emission legislations against rising traffic levels.

Most studies use PM_10_ or PM_2.5_ mass measurements to assess pollutant exposure, as submicron and ultrafine particles are costlier and more difficult to measure. However, the number of particles measured is predominantly found in the submicron range [57]. Using miniaturised scanning mobility particle sizers, Moreno et al. [57]. noted a unimodal distribution of particles with a peak within the range of 30–70 nm in field measurements comparing walking, cycling, cars, buses, trams and subways in Barcelona, Spain. There is, therefore, a need to understand the nature and sources of ultrafine particles in the environments in which populations travel. This work presents the survey measurements of popular travel routes in Bangkok, Thailand, recording the particle number concentrations in a train, underground and overground light railways and a public bus. This work aims to survey the levels of submicron particles that a typical traveller on public transport in Bangkok may be exposed to in order to find the largest sources and provide data to mitigate exposure; these are some of the first presented measurements of sub-micron aerosol concentrations in public transport in Thailand. This study will show how exposure to particulate matter can vary on transport routes and differ from measurements in a fixed location. Differences in particle number concentrations between traffic modes will be evaluated and compared with measurements of pollutants made at a fixed monitoring location. 

## 2. Materials and Methods

Thailand has a tropical climate which has three seasons, two dry seasons (hot and cool) and the rainy season, where monsoon rain is common. The measurements presented here started in the hot season (March 2018) and continued through the rainy season (June–October 2018) with some measurements in the cool season at the end of the year (November 2018). Seasons are often consolidated into dry (hot and cool) and rainy seasons. The temperature of Bangkok is consistently hot, with a yearly average of 28 ± 3 °C in 2018, measured at one of the Thai Pollution Control Department (PCD) stations (Thailand PR Department, lat: 13.783185, long: 100.540489 [58]), with a maximum monthly average of 29 ± 3 °C in June and a minimum of 27 ± 3 °C in January. The rainy season can cause a reduction in particulates due to wash-out [59] but may increase particle production due to increased traffic [26,60]. Cool weather causes a lowering of the boundary layer, trapping pollutants within the city and potentially causing an increase in particle concentrations [31,55]. 

Measurements were taken, when possible, twice in one day to cover the morning and evening rush hours. PNC was measured at a height of approximately 80 cm above ground using a condensation particle counter (TSI 3007) measuring particles of size > 10 nm at a 1 s sampling interval. The TSI particle counter measures particles in sizes from 0.010 to >10 µm and has an upper count limit quoted as 100,000 cm^−3^, above which the instrument may undercount due to coincidence within the optical counter [61]. Measurements of particle count were also made from the 5th floor of the Lak Si building using a Grimm Aerosol Technik 5.403 CPC through an inlet in the window, where samples were taken through a 2 m conducting tube. The Grimm CPC has a measurable size range from 0.0045 to >3 µm and recorded data at 1 Hz. A laboratory comparison was undertaken between both instruments at the start of the campaign and showed good agreement, although the Grimm CPC did measure higher peaks. This is likely to be because it can detect very small newly formed particles which would not be detected by the TSI 3007 CPC (in the size range 4.5–10 nm). More details on these measurements can be found in previous publications [25,26].

To compare PNC data with regional air quality measurements, 6 h averages of CO, NO, NO_2_, Ozone (O_3_), PM_2.5_ and PM_10_ were obtained from the Pollution Control Department (PCD) site at the government PR Department, Bangkok. 

**Figure 1:**
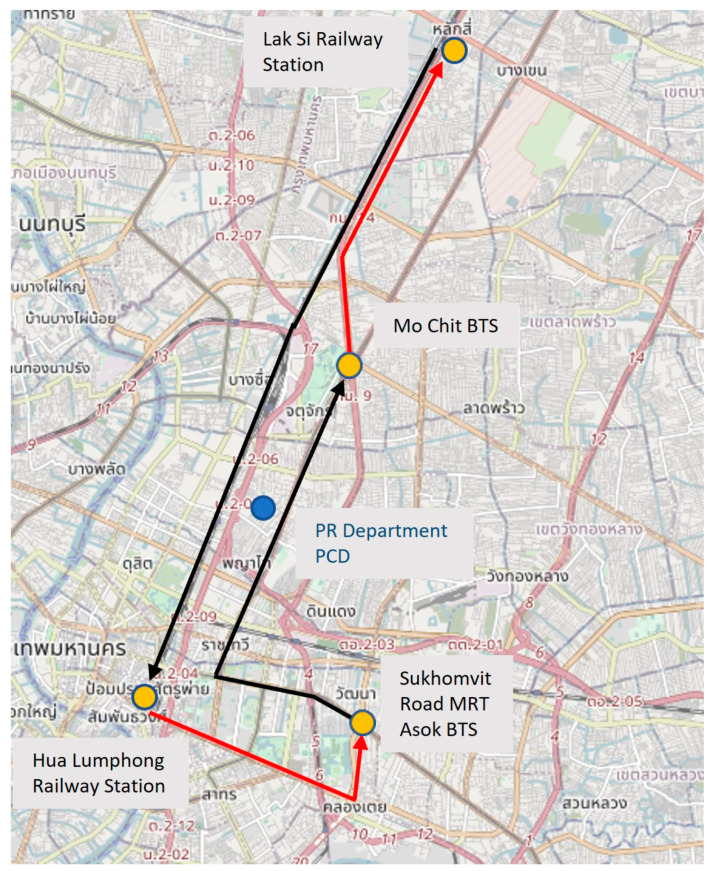
Route taken by train, MRT, BTS and bus through Bangkok. “Map data copyrighted OpenStreetMap contributors and available from https://www.openstreetmap.org”(accessed on 21 March 2023).

A defined route was determined for measurements, which incorporated a number of different transport modes, dates and times of all measurements are shown in Table 1. Each measurement began at the Chulabhorn Research Institute (CRI) with a 600 m roadside walk to the state railway station at Lak Si, then we boarded the train to Hua Lumphong railway station in Bangkok, travelling a journey of 17 km south–west into central Bangkok. Train carriages had open windows and circulated air with fans above. From the rail station, the team walked to the underground MRT station at Hua Lumphong and boarded the MRT train travelling anticlockwise before disembarking at Sukhomvit station, changing to the BTS Sky Train Asok station, then travelling north to Mo Chit. Both MRT and BTS train carriages were sealed units, with ventilation occurring from air conditioning units and periodic opening of the doors at stations. After leaving the MRT, the team walked to the road to board the next air-conditioned bus, travelling 8 km north up Phahonyothin Rd (Asian Highway 1) (1 km), joining Vibhavadi Rangsit (3 km), then Vibhanvadi Rangsit Frontage Road (4 km). The team left the bus outside the Miracle Grand Hotel, walking back the remaining 500 m to the CRI Biomedical building. Nineteen repeats of this route (nine morning, ten afternoon) were made between May 2018 and December 2018. The train, underground, Skytrain and bus routes used for the measurements are shown in Figure 1.

As PNC measured at Lak Si showed a clear diurnal cycle with peaks after 6:00 and 18:00 local time, measurements were chosen to cover the highest exposure time when members of the public were most likely to be travelling. When possible, measurements were taken in the morning and afternoon of a working day in Bangkok. One Sunday (20 May 2018) measurement and one Saturday (27 October 2018) measurement were also included to indicate whether large differences could be expected during the weekend. Traffic in Bangkok is still heavy at the weekend, but slightly reduced; bus journeys are reduced by about 10% at weekends [62], and train services have a reduced timetable [63]. One of the weekday measurements was abandoned due to heavy rainfall which could cause damage to the measuring equipment. It would be expected that PNC on the bus, which shares highways with other road traffic, and the train which travels above ground near to the road network would have peaks at the same time, depending on whether local sources are higher.

## 3. Results and Discussion

**Figure 2:**
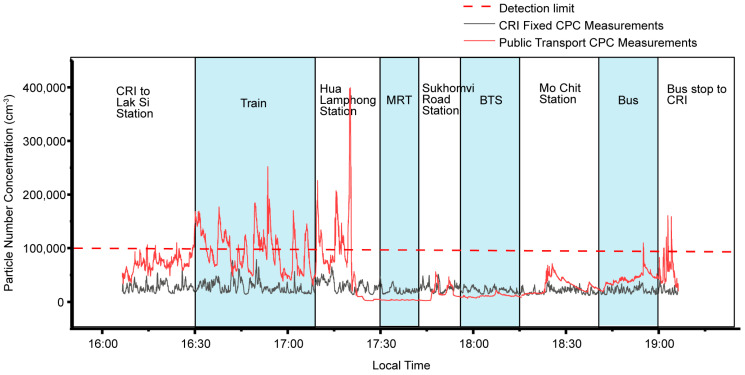
Example time series of particle number concentration on train, MRT, BTS and bus in Bangkok, compared with the particle number concentration measured at a single position. Values above the dotted line are above the limit of detection of the instrument and may be subject to undercounting. Walking and waiting are highlighted in white, while mass transit modes are highlighted in light blue.

Figure 2 shows an example time series from one afternoon measurement on 18 May 2018 compared with the particle count measured from the 5th floor of the CRI lab in Lak Si. The measurements in Lak Si were recorded with an instrument which can detect smaller particles (down to 4.5 nm compared to the TSI CPC at 10 nm). However, the peak size concentration in this experiment was expected to be between 30 and 70 nm [57]. Even with a relative undercount, the TSI measurements on the transport route are higher than the fixed measurement, indicating the importance of proximity to the source. Walking routes between transport modes are shown between transport time series. Typically, handheld measurements at roadside are more variable than the fixed site (16:00–16:30 LT and 19:00–19:10 LT). The train shows the most changeable time series, frequently stopping and restarting on its journey. The largest peak in this example time series was at 17:15 LT when walking along the train platform and passing the engines. Many of the peaks in this example on the state railway journey, and in walks near to the railway, are higher than the 100,000 cm^−3^ limit of the CPC and may be subject to undercounting. There are step changes in particle concentrations as the measurements travelled to the underground station at Hua Lumphong, which has two lower levels. The particle concentration is lowest in the lowest MRT station and on the MRT. Within the BTS and bus measurements, particle concentrations are elevated as doors open to let passengers in, but then decay during the journey.

**Figure 3:**
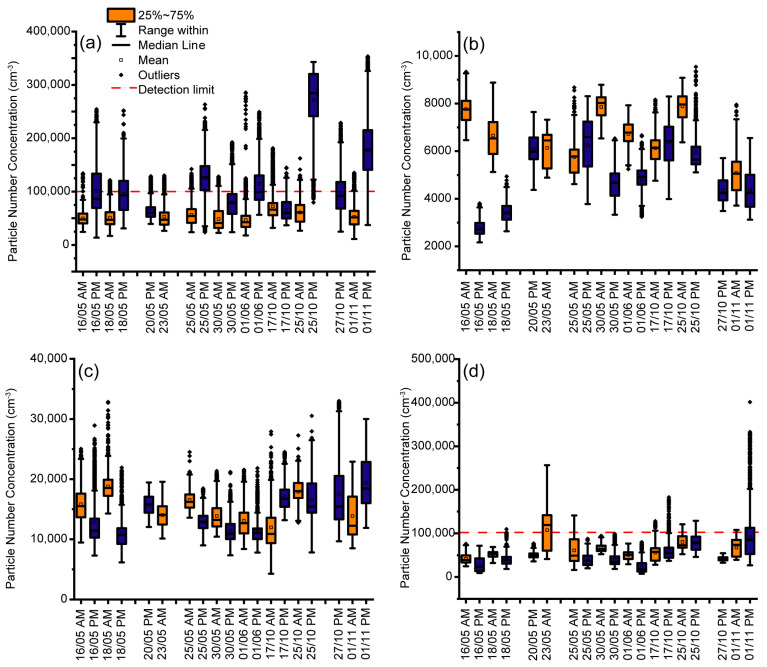
Box plots showing particle counts measured on public transport in the morning (orange) and evening (blue) on the selected dates of May–June, October–November, 2018. Values above the dotted line are above the limit of detection of the instrument and may be subject to undercounting. Most of the measurements are part of a pair performed on the same day, gaps represent a morning or afternoon when measurements could not be performed. Particle counts are shown on (**a**) the state railway train, (**b**) the MRT underground, (**c**) the BTS Skytrain and (**d**) the public bus.

The results of all journeys are summarised in Figure 3, with morning and afternoon measurements paired together. The total estimated mean PNC (and standard deviation) of each journey mean for all train, MRT, BTS and bus journeys were 83,000 ± 5800 cm^−3^, 57,000 ± 1400 cm^−3^, 15,000 ± 3000 cm^−3^ and 57,000 ± 22,000 cm^−3^ with maximums of 9500 cm^−3^ and 33,000 cm^−3^ on the MRT and BTS, respectively, while the train and bus had maximums above 100,000 cm^−3^. The particle counter used has an upper limit of 100,000 cm^−3^ before coincidence within the optical detector, causing undercounting, but an estimate can be used to account for this [61]. The correction, if applied, does not affect the BTS or MRT mean or maximum, but increases the mean of all train journeys to 95,000 cm^−3^ with a maximum of 505,000 cm^−3^ and increases some of the bus outliers, with the maximum value at 746,000 cm^−3^, but with only a negligible difference to the mean. It was only possible to include one measurement on a Saturday and one measurement on a Sunday, but the mean concentrations on those days were mostly within 1 standard deviation of the mean of all measurements, and so it is not clear with this small sample that there would be any significant difference on these days. 

The monitoring of ultrafine particles was recommended in enclosed railway stations by Thornes et al. [46] and the results presented here suggest that measurements should also be extended to platforms where peak concentrations can reach very high levels. The underground MRT system had much lower concentrations than both bus and train travel, and this is similar to a study in Taipei which showed PNC in their underground system to be close to, or less than, background levels [64]. However, larger particles such as in PM_2.5_ may still be produced by abrasion within the system, as has been seen in older networks [65,66,67]. Low concentrations of BTEX were found on the Skytrain when compared with buses in another study in Bangkok; this is in agreement with the low PNC measurements presented here [68].

The MRT and BTS are used by more passengers than the state railway and bus, but as the distance between stops is longer in the state railway, it may be assumed that travellers by train may have a longer exposure time to high levels of pollutants.

To aid understanding of where pollutants come from and where points of higher exposure may be, the time series can be investigated. In many journeys, the time series of each transport mode included clear peaks which were followed by a decay back to baseline, assumed to arise from the opening of doors at stops and the influx of higher levels of particles from outside the mode of transport. It was possible to calculate a time constant for the decay half-life from decays for some of these journeys. Table 2 shows the calculated half-life of decay in each transport mode. It is impossible to travel on the exact same vehicle within the mode of transport, and so there will be some variability; in addition, other factors such as temperature and humidity can affect decay rates. However, the half-lives are very similar for each mode of transport; with uncertainty, there is no clear difference, with an estimate of 2–3 min as a mean value. A half-life of around 2–3 min typically does have implications for the exposure of passengers, as high initial concentrations on door opening, particularly at ‘hot spot’ locations, mean that passengers are exposed to elevated (PNC maximum approximately 350,000, 9000, 33,000 and 330,000 cm^−3^ for train, MRT, BTS and bus, respectively) levels for potentially several minutes. The time series analyses suggest that the majority of exposure to particulate matter for these passengers occurs when the mode of transport stops and allows passengers on or off. There may also be important implications for the spread of a variety of airborne pathogens, e.g., COVID-19. Although impossible to estimate with any certainty, the data reported by Zuurbier et al. [39] suggest half-lives of around 2–5 min for both diesel and electric buses, which are consistent with the data presented here.

To test whether there was a significant difference between particle counts measured in the morning or the afternoon, a two-tailed Students t-test was performed on the means of PNC for the measurements in the train, MRT, BTS and bus. Using data uncorrected for the undercounts above 100,000 cm^−3^, there was a significant (*p* < 0.05) difference for the train measurements, with the mean of all mean PM measurements of 107,921 ± 71,411 in the afternoon and 54,633 ± 7871 in the morning. This is in contrast to particle measurements at a fixed location [25,26] which showed particle numbers at their highest during the morning rush hour. Conversely, within the MRT, there was a smaller but significant (*p* < 0.01) difference in PNC, which was higher in the morning (6664 ± 1002) than the afternoon (4909 ± 1238). BTS and bus did not show a significant difference. There are two potential reasons why the train might have higher PNC in the afternoon compared to the morning, and further investigations would be required to determine which is more likely. It was shown in recent studies that PNCs are higher when the engine is working to pull the train as opposed to pushing [47,48,49,50]. It may be that the timetabling of the state railway in Bangkok biases more toward push trains in the morning than pull trains. The length of journey may also be an important consideration, although journey lengths are highly variable, with 49 min on average in the morning and 76 min on average in the afternoon. A slower journey may involve travelling at slower speeds, but also might include extended wait times and more acceleration and deceleration—each of which might affect the output of the diesel engine, for example, through particle production by mechanical braking [69] or by increased fuel usage during acceleration [50]. 

Rain has the potential to influence PNC by causing washout, reducing particle concentration or conversely increasing traffic sources as the traffic volume increases during rainy periods in Bangkok [59,60]. The difference between dry and rainy seasons was analysed using a two-tailed Student’s t-test, but no significant difference was shown. However, measurements in the rainy season were postponed or cancelled when there was particularly heavy rainfall, which might otherwise have altered the mean concentrations observed.

**Figure 4:**
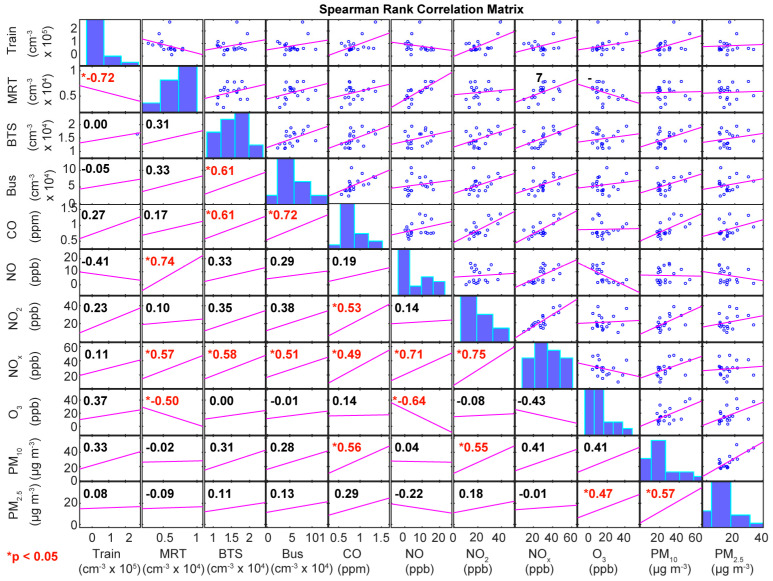
Spearman’s rank correlation matrix of average particle concentrations measured on the train, MRT, BTS and bus alongside contemporaneous 5 h averages of pollutants measured at the PCD at the PR department, central Bangkok. The top right panels show the scatter plot of the pair ov values and purple lines represent a least squares reference line, numbers in the bottom left half indicate the Spearman rank correlation coefficient and red numbers (with asterisks)indicate significant correlations (*p* < 0.05). The bar charts in the central diagonal represent the distribution of the variable as a histogram.

The background air quality was measured at several PCD sites across Bangkok. Averages of CO, NO, NO_2_, O_3_, PM_10_ and PM_2.5_ were measured at one site for the 5 h duration of each measurement to test whether pollutants measured on the transport modes were related to background pollutants. As traffic is known as a major source of PNC [23] and traffic is also the major source of CO in a city, a relationship between CO and PNC would be expected if city-wide sources were more important than local sources. The number concentration was measured in this study, while PM_2.5_ and PM_10_ are a measurement of mass; however, if particles have a common source, a relationship might be expected. If local sources are more important, then correlations would be weak. Spearman’s rank correlation analysis was chosen as many air quality data are non-parametric, and ranked data will remove any inaccuracies introduced by the undercounts above 100,000 particles cm^−3^. There are weak significant (*p* < 0.05) positive correlations between CO measured at the PCD site, and PNC measured in the BTS and bus (Figure 4), indicating that city-wide traffic may contribute to the level of particles in these places. There is also a very strong correlation between NO measured at the PCD site and PNC measured underground in the MRT, which was unexpected. Air is brought into the stations from the ground level, which may contain NO, and would then have an elongated lifetime below ground. If PNC is also related to above-ground concentrations, then some correlation may be expected. A mass concentration of PM_10_ and PM_2.5_ did not correlate with PNC in any transport mode, which is consistent with the majority of particulate mass being found in larger particles [23]. The interactions between meteorology and air chemistry in urban air is a complex problem that is ill-described by simple correlations, so it is important to consider the limit of this analysis. It can be concluded that there is a strong decoupling of local PNCs in these transport modes to background measurements in the city, and therefore, local sources are of most importance.

Currently, trains and buses in Bangkok use diesel. Thailand is increasing its capacity for electric trains and is expecting to replace its diesel lines in Bangkok with electric lines. The addition of more light railways, similar to the MRT and BTS system, could reduce passenger exposure to ultrafine particles. There are also plans to change diesel fuel by moving towards biofuel mixtures, which is likely to change the amount of pollutants produced [70]. Jedynska et al. [71] measured a decrease in PNC produced with biofuel mixtures per brake-specific fuel consumption. Diesel (B0) produced 0.1 g kWh^−1^, while B10 and B20 reduced that to 0.09 and 0.07 g kWh^−1^, respectively. While the particulates measured on these transport modes could be from multiple sources, this would represent a significant reduction in primary particle production.

It is clear that there are places, such as railway station platforms, where there can be very high peaks in particle concentrations, and should be avoided by vulnerable people. The data show how important ventilation conditions can be and the effect of opening doors in polluted areas; it may be worth considering where and when buses collect and drop off passengers, as if it is possible to do so away from the main roads, exposures may be reduced. 

Mask-wearing to protect against high particulate pollution is common in some countries. While known to reduce exposure to airborne pathogens [72], their effectiveness against pollutants, in particular gases and ultrafine particles, may be limited. Huang and Morawska [73] questioned their effectiveness against pollutants, as masks do not protect against pollutant gases and may engender a false sense of security. For a given mask design and material, the filtration of particles is dependent on the size of particles [74]. More detailed analysis of the nature of the particles would be required for further recommendations, as it is not apparent from these results what the size distributions of the particles are in each source. It is possible that a high number of submicron or ultrafine particles exist in roadside sources, as shown by roadside measurements in the same study area [26], which may not be effectively removed by typically worn face coverings.

## 4. Conclusions

There is a need to understand the health risks of particles in the submicron-size range [20,21]; due to the heterogenic nature of submicron particles in cities, this requires personal monitoring [22]. A significant proportion of travellers in Bangkok use public transport including the state railway, MRT underground system, BTS Skytrain and bus network, and the number of journeys is increasing. These measurements are among the first in Bangkok transport systems and have shown very different particle counts with PNC in the region of 100,000 cm^−3^ on the train and bus, but lower than 20,000 cm^−3^ and 10,000 cm^−3^ on the MRT and BTS, respectively. The train and bus concentrations were higher than at a corresponding fixed measurement site in Lak Si, Bangkok. Bangkok is currently upgrading its public transport network to include more MRT and Skytrain systems. It is likely that these will reduce occupant exposure to PNC compared with the existing state railway.

This study is limited in its scope and would benefit from follow-up studies that are able to ascertain the reasons behind the high particle number concentrations, particularly on the state railway. The high concentration observed in some transport modes and at roadsides was sometimes above the upper concentration limit of the CPC in use and may represent an undercount, which could potentially be addressed by using different instrumentation. In addition, any future studies of the toxicological effects of PNC would benefit from sampling and subsequent chemical analysis of the airborne particles present in each area and/or mode of transport.

## Figures and Tables

**Table 1:** Dates and times of measurements through the state railway (Train), Mass Rapid Transport underground system (MRT), BTS overground Skytrain (BTS) and Bus system. Times between ‘Start’ and ‘Finish’ are not included within each of the named transport modes which involved walking between different transport stations.

Day	Date	Start Time	Train	MRT	BTS	Bus	Finish	Season
Wed AM	16 May 2018	06:34	06:48–07:40	07:54–08:05	08:13–08:34	08:40–09:00	09:08	Hot
Wed PM	16 May 2018	16:17	16:27–17:17	17:27–17:38	17:44–18:10	18:20–18:58	19:04	Hot
Fri AM	18 May 2018	16:34	06:45–08:02	08:12–08:22	08:31–08:52	09:02–09:21	09:28	Hot
Fri PM	18 May 2018	16:06	16:30–17:18	17:30–17:41	17:54–18:15	18:24–18:59	19:06	Hot
Sun PM	20 May 2018	17:00	17:12–17:58	18:13–18:23	18:32–18:53	19:02–19:21	19:27	Hot
Wed AM	23 May 2018	06:34	06:45–07:41	07:50–08:01	08:11–08:33	08:43–09:05	09:11	Hot
Fri AM	25 May 2018	06:21	06:30–07:44	08:12–08:33	08:41–09:01	09:10–09:34	09:41	Hot
Fri PM	25 May 2018	16:11	16:26–17:42	17:53–18:06	18:20–18:43	18:55–19:19	19:26	Hot
Wed AM	30 May 2018	07:03	07:26–08:46	08:55–09:04	09:25–09:51	10:05–10:22	10:30	Rainy
Wed PM	30 May 2018	16:10	16:34–16:40	17:38–17:50	17:59–18:24	18:30–19:14	19:20	Rainy
Fri AM	1 June 2018	06:31	06:50–08:05	08:14–08:26	08:36–08:59	09:07–09:28	09:36	Rainy
Fri PM	1 June 2018	16:09	16:27–17:08	17:19–17:30	17:41–18:03	18:20–19:07	19:13	Rainy
Wed AM	17 October 2018	06:35	06:44–07:57	08:14–08:25	08:33–08:55	09:09–09:25	09:32	Rainy
Wed PM	17 October 2018	16:20	17:14–18:02	18:18–18:30	18:42–19:06	19:22–19:43	19:51	Rainy
Thu AM	25 October 2018	07:08	07:27–08:28	08:43–08:54	09:04–09:30	09:44–10:01	10:09	Rainy
Thu PM	25 October 2018	16:20	16:29–17:12	17:31–17:43	17:56–18:19	18:34–19:06	19:15	Rainy
Sat PM	27 October 2018	14:58	15:30–16:02	17:28–17:40	17:50–18:12	18:28–18:46	18:53	Rainy
Thu AM	1 November 2018	06:33	06:42–08:31	08:47–08:58	09:12–09:32	09:44–09:59	10:07	Cool
Thu PM	1 November 2018	16:15	16:26–17:11	17:26–17:38	17:51–18:15	18:35–19:20	19:21	Cool

**Table 2:** Half-lives (in minutes) determined in the time series of PNC on four transport modes, from a total of 19 analysed time series.

	Number	Minimum	Maximum	Mean	SD
Train	12	0.7	3.6	2.1	1.0
MRT	11	1.3	5.7	2.5	1.3
BTS	11	0.5	3.6	1.7	1.0
Bus	13	0.8	4.5	2.8	1.0

## Data Availability

Data will be deposited in the CEDA project Ultrafine and Submicron Particles in the Urban Environment in Thailand–Size, Concentration, Composition and Health Impacts. https://catalogue.ceda.ac.uk/uuid/d753d76d68e946baa19484b2307b7748.
